# Application of ordinal logistic regression analysis to identify the determinants of illness severity of COVID-19 in China

**DOI:** 10.1017/S0950268820001533

**Published:** 2020-07-07

**Authors:** Kandi Xu, Min Zhou, Dexiang Yang, Yun Ling, Kui Liu, Tao Bai, Zenghui Cheng, Jian Li

**Affiliations:** 1 Department of Respiration and Critical Care Diseases, Ruijin Hospital, School of Medicine, Shanghai Jiao Tong University, Shanghai, China; 2 Institute of Respiratory Diseases, School of Medicine, Shanghai Jiaotong University, Shanghai, China; 3 Department of Respiratory Diseases, Tongling People's Hospital, Tongling, China; 4 Department of Infectious Disease, Shanghai Public Health Clinical Center, Shanghai, China; 5 Department of Respiratory and Critical Care Medicine, Tongji Medical College of Huazhong University of Science and Technology, Wuhan, China; 6 Department of Infectious Disease, Wuhan Jinyintan Hospital, Wuhan, China; 7 Department of Radiology, Ruijin Hospital, School of Medicine, Shanghai Jiao Tong University, Shanghai, China; 8 Clinical Research Center, Ruijin Hospial, School of Medicine, Shanghai Jiao Tong University, Shanghai, China

**Keywords:** COVID-19, determinant, ordinal logistic regression, severity

## Abstract

Corona Virus Disease 2019 (COVID-19) has presented an unprecedented challenge to the health-care system across the world. The current study aims to identify the determinants of illness severity of COVID-19 based on ordinal responses. A retrospective cohort of COVID-19 patients from four hospitals in three provinces in China was established, and 598 patients were included from 1 January to 8 March 2020, and divided into moderate, severe and critical illness group. Relative variables were retrieved from electronic medical records. The univariate and multivariate ordinal logistic regression models were fitted to identify the independent predictors of illness severity. The cohort included 400 (66.89%) moderate cases, 85 (14.21%) severe and 113 (18.90%) critical cases, of whom 79 died during hospitalisation as of 28 April. Patients in the age group of 70+ years (OR = 3.419, 95% CI: 1.596–7.323), age of 40–69 years (OR = 1.586, 95% CI: 0.824–3.053), hypertension (OR = 3.372, 95% CI: 2.185–5.202), ALT >50 μ/l (OR = 3.304, 95% CI: 2.107–5.180), cTnI >0.04 ng/ml (OR = 7.464, 95% CI: 4.292–12.980), myohaemoglobin>48.8 ng/ml (OR = 2.214, 95% CI: 1.42–3.453) had greater risk of developing worse severity of illness. The interval between illness onset and diagnosis (OR = 1.056, 95% CI: 1.012–1.101) and interval between illness onset and admission (OR = 1.048, 95% CI: 1.009–1.087) were independent significant predictors of illness severity. Patients of critical illness suffered from inferior survival, as compared with patients in the severe group (HR = 14.309, 95% CI: 5.585–36.659) and in the moderate group (HR = 41.021, 95% CI: 17.588–95.678). Our findings highlight that the identified determinants may help to predict the risk of developing more severe illness among COVID-19 patients and contribute to optimising arrangement of health resources.

## Introduction

The pandemic of the novel corona virus disease 2019 (COVID-19), which originally emerged in Wuhan, China in December 2019 has spread around the world [1, 2]. As of 5 June 2020, the WHO has reported a total of 6 535 354 COVID-19 cases and 387 155 deaths globally, with an average mortality of 5.92% and the person-to-person transmission is still continuing [3]. The clinical spectrum of COVID-19 appears to be wide, ranging from asymptomatic infection to mildly, severely, critically ill cases. Mild patients present only upper respiratory tract symptoms like cough and fever, however, respiratory failure, acute respiratory distress syndrome, heart failure, septic shock and even death can be observed in patients with critical conditions [4]. Although most confirmed patients (81%) were classified as mild or moderate, 14% were severe and 5% were critical according to the largest investigation of 72 314 cases to date [5]. Accumulated evidences have indicated that older age, male, smoking, comorbidity, neutrophilia, coagulopathy, elevated D-dimer level and organ dysfunction were associated with increased risk of death from COVID-19 [5–10]. However, investigations of determinants of severity of COVID-19 are scarce. Early detecting cases with the potential deterioration of disease will contribute to optimising the use of limited health resources and allocating the proper care. Liang *et al*. developed a clinical risk score to predict the occurrence of critical COVID-19 based on severe or non-severe [11]. To our knowledge, no previous studies have been conducted to investigate the risk factors of severity of COVID-19 based on ordinal response, namely moderate, severe and critical illness. The estimation of risk factors of disease severity is therefore not very robust.

Here, we conducted a retrospective study based on COVID-19 patients from four designated hospitals in Shanghai, Hubei and Anhui provinces to describe the clinical features of COVID-19, and aimed to identify the predictors of multi-level response of severity from moderate, severe to critical illness.

## Methods

### Study design and participants

This multi-centre retrospective study encompassed COVID-19 patients classified as being moderately, severely and critically ill. The illness severity of COVID-19 was defined according to the Guideline on the Diagnosis and Treatment of COVID-19 by the National Health Commission (V.5) as described previously [12]. Patients were admitted to Shanghai Public Health Clinical Center, Wuhan Jinyintan Hospital and Tongji Hospital of Tongji Medical College HUST in Hubei province and Tongling Municipal People's Hospital in Anhui province from 1 January 2020 to 8 March 2020. All patients recruited in this study were laboratory-confirmed COVID-19. The study was approved by the Ethics Committees of these four hospitals, respectively. Written informed consent was waived owing to the need of rapid emergency response to this infectious disease.

### Data collection

Medical records of COVID-19 patients were reviewed by the research team, and demographic, epidemiological, clinical, laboratory, treatment and outcome data were retrieved from electronic medical records using a standardised case report form. All data were cross-checked by two experienced doctors. To ascertain the medical histories or epidemiological data, which were unavailable from electronic medical records, the patients or their close relatives were interviewed by researchers. Data from the medical records were adopted if there was a discrepancy between the subjective description and the medical records.

### Laboratory procedures

Method of laboratory confirmation of severe acute respiratory syndrome coronavirus 2 (SARS-CoV-2) has been described elsewhere [1]. Simply, the Chinese Center for Disease Control and Prevention (CDC) and local CDC were in charge of detecting SARS-CoV-2 in throat-swab specimens from the upper respiratory tract by real-time reverse transcription polymerase chain reaction assay (RT-PCR). The criteria of discharge included absence of fever for at least 3 days, remission of respiratory symptoms, complete improvement in bilateral lungs in chest CT, together with negative for 2 times in throat-swab samples for SARS-CoV-2 RNA at least 24 h apart.

Initial clinical laboratory examinations involved complete blood count, serum biochemical tests (including liver and kidney functions, creatine kinase, lactate dehydrogenase (LDH) and electrolytes), myocardial enzymes, D-dimer and procalcitonin (PCT). Frequency of examinations was under the discretion of treating physicians. Chest computed tomographic (CT) scans were carried out for all COVID-19 patients. Two radiologists were invited to interpret chest CT scans independently and were blinded to the severity of the patient. When disagreement arose, a third radiologist was consulted to reach a final decision.

### Statistical analysis

Continuous variables were presented as median with interquartile range (IQR) and the analysis of variance (ANOVA) or Kruskal−Wallis *H* test were used to compare the difference among three groups as appropriate. Categorical variables were expressed as frequency with percentages, and were analysed by Pearson's *χ*^2^ test or Fisher's exact test. Bonferroni's correction was used for pairwise comparison. All patients were divided into moderate, severe and critical illness groups. Potential predictive variables included the following case characteristics on admission: demographic and epidemiological features, comorbidity, clinical signs and symptoms, laboratory findings and chest imaging results. To explore the risk factors associated with illness severity of COVID-19, namely moderately, severely and critically ill, which means the response variable was ordinally scaled, a cumulative logit model was used to investigate the effect of predictors of COVID-19 severity. Imputation for missing variables of some patients at hospital admission was considered if missing values were less than 20%, and imputation based on the expectation−maximisation algorithm method was used to replace missing values. Before ordinal logistic regression model was fitted, continuous variables of laboratory findings were transformed into categorical variables according to their reference values. The univariate and multivariate cumulative logit models were fitted with moderate illness as the reference level. Potential predictors of severity were investigated using univariate ordinal logistic regression firstly. We further conducted a backward stepwise multivariate ordinal logistic regression analysis excluding variables which were not significant in univariate cumulative logit model. Since missing rate of 34.6% occurred in the lung imaging results and over 40% existed in urine protein and urine glucose, these variables were excluded from multivariate ordinal logistic model. The overall survival (OS) was estimated using the method of Kaplan−Meier and the log-rank test was applied to compare the survival difference among different severity illness groups. The hazard ratio with 95% confidence interval (CI) was estimated with Cox proportional hazard model. A two-sided *α* of less than 0.05 was considered statistically significant. All statistical analyses were conducted using SAS software (V. 9.4) (SAS Institute Inc., USA).

## Results

### Demographics, laboratory findings and clinical course

As of 28 April 2020, data from 598 COVID-19 cases admitted to these four hospitals, including 400 (66.89%) moderate cases, 85 (14.21%) severe cases and 113 (18.90%) critical cases, had been collected to be incorporated into this study, of whom 79 cases had died during hospitalisation, with an average mortality of 13.21%, and 457 cases had recovered and been discharged. The remaining 62 cases were still in hospitals. The median age of the 598 patients was 57 years (IQR 42–66), ranging from 11 to 89 years, and 58.03% patients were male (Table 1). At least one comorbidity was present in 51.54% of patients, with hypertension being the most frequent comorbidity (33.90%), followed by diabetes (13.18%) and cardiovascular disease (6.51%). Few cases had a current (7.53%) or former (2.57%) smoking habit. The most common symptoms on admission were fever (81.22%) and dry cough (33.63%), followed by sputum production (30.77%) and shortness of breath (26.65%). Overall, the median interval between illness onset and confirmed diagnosis was 4 days (IQR 2–7), whereas the median interval between illness onset and admission was 7 days (IQR 3–11).

**Table 1.** Clinical characteristics and comorbidities of 598 COVID-19 patients

Variable	All (*n* = 598)	Illness Severity			*P* value
		Moderate (*n* = 400)	Severe (*n* = 85)	Critical (*n* = 113)	
Age (years)	57 (42–66)	52 (39–64)*†	61 (49.5–67)*	65.5 (58.25–72)	<0.0001
Sex					0.067
Male	347/598 (58.03)	219/400 (54.75)	54/85 (63.53)	74/113 (65.49)	
Female	251/598 (41.97)	181/400 (45.25)	31/85 (36.47)	39/113 (34.51)	
Smoking					0.883
Never	525/584 (89.90)	355/399 (88.97)	74/85 (87.06)	91/100 (91.00)	
Current	44/584 (7.53)	33/399 (8.27)	4/85 (4.71)	7/100 (7.00)	
Former	15/584 (2.57)	11/399 (2.76)	2/85 (2.35)	2/100 (2.00)	
Symptoms					
Fever (temperature ⩾37.3°C)	454/559 (81.22)	324/400 (81.00)	70/85 (82.35)	60/74 (81.08)	0.958
Dry cough	188/559 (33.63)	120/400 (30.00)*	43/85 (50.59)	25/74 (33.78)	0.001
Sputum production	172/559 (30.77)	120/400 (30.00)	22/85 (25.88)	30/74 (40.54)	0.112
Pharyngodynia	38/559 (6.80)	31/400 (7.75)	6/85 (7.06)	1/74 (1.35)	0.132
Chest pain	28/559 (5.01)	18/400 (4.50)	5/85 (5.88)	5/74 (6.76)	0.581
Shortness of breath	149/559 (26.65)	59/400 (14.75)*†	59/85 (69.41)*	31/74 (41.89)	<0.0001
Any comorbidity	301/584 (51.54)	167/399 (41.85)	40/85 (47.06)	94/100 (94.00)	<0.0001
Hypertension	198/584 (33.90)	91/399 (22.81)*†	31/85 (36.47)*	76/100 (76.00)	<0.0001
Cardiovascular disease	38/584 (6.51)	25/399 (6.27)	4/85 (4.71)	9/100 (9.00)	0.469
Diabetes	77/584 (13.18)	42/399 (10.53)*	7/85 (8.24)*	28/100 (28.00)	<0.0001
Carcinoma	21/584 (3.60)	11/399 (2.76)*	2/85 (2.35)	8/100 (8.00)	0.048
Cerebrovascular disease	19/584 (3.25)	13/399 (3.26)	0/85 (0.00)	6/100 (6.00)	0.052
COPD	11/584 (1.88)	7/399 (1.75)	2/85 (2.35)	2/100 (2.00)	0.809
Others	99/584 (16.95)	86/399 (21.55)*†	6/85 (7.06)	7/100 (7.00)	<0.0001
Days from illness onset to diagnosis confirmed	4 (2−7)	4 (2−6)	8 (5−13)*	7 (4−11)	<0.0001
Days from illness onset to admission	7 (3−11)	6 (3−10)*†	10 (6−14)	10 (0−14)	<0.0001

COPD, chronic obstructive pulmonary disease.

Data are median (IQR) or *n*/ total (%). *P* value denotes the comparison among moderate, severe and critical illness group. * and †Signify *P* < 0.05 for pos-hoc comparison. *Refers to comparison between the critical group and the severe group or the moderate group. †Refers to comparison between the severe group and the moderate group.

The substantial differences of laboratory findings on admission among three groups of patients were observed (Table 2). The results indicated that significantly higher proportion of patients showing abnormal alanine aminotransferase (ALT) and aspartate aminotransferase (AST), and significantly higher levels of C-reactive protein, neutrophil count, serum potassium, cardiac troponin I (cTnI), myohaemoglobin, PCT, brain natriuretic peptide (BNP) and D-dimer were observed in the critically ill group and the severely ill group than in the moderately ill group, whereas, the levels of haemoglobin and serum albumin were significantly lower in critically ill group and severely ill group as compared with moderately ill group (*P* < 0.05). The critical group showed a significantly higher level of platelet count, fibrinogen and serum calcium as compared with severe group and moderate group. The severe group showed a significantly lower level of lymphocyte count than the moderate group. The normal ranges of laboratory indicators are shown in Supplementary Table S1. Abnormalities on chest radiographs on admission were seen in most patients (Table 2). Overall, typical findings on chest CT images were ground-glass opacity (94.37%), followed by pleural thickening (47.06%) and consolidation (35.74%). Chest CT scans showed significantly higher percentage of bilateral lungs involvement in critical group (92.45%) and severe group (95.24%) as compared with moderate group (80.07%). The median lung lobes involved in critical group (5, IQR 5–5) and severe group (5, IQR 2.75–5) were greater than those in moderate group (4, IQR 2–5).

**Table 2.** Laboratory and chest CT findings on admission of 598 COVID-19 patients

Variable	All (*n* = 598)	Disease severity			*P* value
		Moderate (*n* = 400)	Severe (*n* = 85)	Critical (*n* = 113)	
Laboratory findings					
C-reactive protein (mg/l)	18.80 (5.17–60.22)	15.40 (4.85–42.98)*†	63.05 (18.55–159.43)	37.00 (3.95–129.35)	<0.0001
White blood cell count (×10^9^/l)	5.51 (4.38–7.52)	5.22 (4.20–6.63)*	7.03 (4.62–10.21)	6.26 (4.91–8.76)	<0.0001
Neutrophil count (×10^9^/l)	3.59 (2.70–5.37)	3.40 (2.54–4.69)*†	5.27 (3.37–8.94)	3.68 (3.09–5.53)	<0.0001
Lymphocyte count (×10^9^/l)	1.03 (0.69–1.48)	1.10 (0.76–1.49)†	0.86 (0.60–1.18)	0.94 (0.57–1.49)	0.002
<1.0 × 10^9^/l	285/598 (47.66)	166/400 (41.5) †	58/85 (68.24)	61/113 (53.98)	<0.0001
Haemoglobin (g/l)	132 (120–143)	134 (123–145)*†	129 (114–138)	127 (113–136)	<0.0001
Platelet count (×10^9^/l)	188 (146–253)	185 (145–238)*	188 (144–240)*	274 (178–338)	<0.0001
ALT >50 μ/l	136/598 (22.74)	60/400 (15.0) *†	23/85 (27.06) *	53/113 (46.90)	<0.0001
AST >40 μ/l	174/598 (29.10)	82/400 (20.50) *†	33/85 (38.82)	59/113 (52.21)	<0.0001
Total bilirubin (μmol/l)	9.8 (7.5–13.8)	9.7 (7.3–14.0)	10.7 (8.8–14.8)*	9.0 (7.2–13.0)	0.025
Direct bilirubin (μmol/l)	3.9 (3.1–5.6)	4.0 (3.0–5.7)	4.0 (3.1–5.5)	3.9 (3.1–5.9)	0.654
Albumin (g/l)	36.94 (32.48–41.32)	38.88 (34.55–42.40)*†	31.90 (28.73–34.28)*	34.05 (31.48–37.58)	<0.0001
Blood urea nitrogen (mmol/l)	4.80 (3.70–5.90)	4.75 (3.66–5.82)	5.10 (3.80–6.35)	4.80 (4.10–5.80)	0.804
Creatinine (μmol/l)	68.12 (55.00–81.05)	65.51 (52.93–78.27)*	70.30 (57.00–82.50)	76.00 (62.25–90.50)	<0.0001
Urinary glucose					0.956
Negative	268/325 (82.46)	184/222 (82.89)	48/59 (81.36)	36/44 (81.82)	
Positive	57/325 (17.54)	38/222 (17.11)	11/59 (18.64)	8/44 (18.18)	
Urinary protein					0.087
Negative	224/343 (65.31)	150/222 (67.57)	44/64 (68.75)	30/57 (52.63)	
Positive	119/343 (34.69)	72/222 (32.43)	20/64 (31.25)	27/57 (47.37)	
Sodium (mmol/l)	139 (137–141)	139 (137–141)†	141 (138––142)*	139 (136–142)	0.007
Potassium (mmol/l)	3.9 (3.6–4.3)	3.9 (3.6–4.1)*†	4.3 (3.8–4.7)	4.2 (3.8–4.5)	<0.0001
Calcium (mmol/l)	2.07 (1.98–2.17)	2.05 (1.97–2.16)*	2.04 (1.94–2.14)*	2.16 (2.08–2.25)	<0.0001
LDH (μ/l)	264 (207–376)	242 (197–326)	345 (238–479)	320 (249–497)	0.767
Creatine kinase (μ/l)	82 (54–151)	79 (53–137)	96 (55–184)	81 (53–198)	0.619
CK-MB (μ/l)	9.97 (13.00–17.00)	12.94 (10.41–16.31)*	13 (10.00–18.00)	10.00 (0.78–19.25)	0.035
Myohaemoglobin (ng/ml)	24.51 (5.76–59.80)	13.41 (3.91–41.30)*†	61.55 (33.10––95.70)	67.40 (32.40–160.10)	<0.0001
cTnI (ng/ml)	0.07 (0.02–5.00)	0.02 (0.01–0.07)*†	4.85 (2.00–15.13)	6.49 (2.80–19.68)	<0.0001
PCT (μg/l)	0.05 (0.02–0.09)	0.03 (0.02–0.06) *†	0.06 (0.05–0.18)	0.08 (0.05–0.24)	<0.0001
ESR (mm/h)	49 (29–79)	47 (27–83)	51 (35–72)	52 (29–68)	0.638
BNP (pg/ml)	46.00 (24.00–101.00)	36.00 (22.00–72.50)*†	61.00 (34.00–150.00)	106.00 (52.80–260.00)	<0.0001
Fibrinogen (g/l)	4.60 (3.72–5.81)	4.30 (3.62–5.27)*	5.20 (3.44–6.20)*	6.49 (4.86–11.48)	<0.0001
D-dimer (μg/l)	0.61 (0.35–1.39)	0.50 (0.32–0.99)*†	0.98 (0.56–3.14)	1.07 (0.58–2.65)	<0.0001
Chest CT findings					
Lung lobes involved	4 (2–5)	4 (2–5)*	5 (2.75–5)	5 (5–5)	<0.0001
Bilateral lungs involved	326/391 (83.38)	237/296 (80.07)*†	40/42 (95.24)	49/53 (92.45)	0.008
Consolidation	104/391 (35.74)	91/296 (30.74)†	3/42 (7.14)	10/53 (18.87)	0.002
Ground-glass opacity	369/391 (94.37)	277/296 (93.58)	42/42 (100.00)	48/53 (90.57)	0.123
Linear opacity	94/391 (24.04)	62/296 (20.94)†	20/42 (47.62)*	12/53 (22.64)	0.001
Pleural effusion	22/391 (5.63)	16/296 (5.41)	3/42 (7.14)	3/53 (5.67)	0.866
Pleural thickening	184/391 (47.06)	149/296 (50.34)†	13/42 (30.95)	22/53 (41.51)	0.043

ALT, alanine aminotransferase; AST, aspartate aminotransferase. LDH, lactate dehydrogenase; CK-MB, creatine kinase isoenzymes; cTnI, cardiac troponin I; PCT, procalcitonin; ESR, erythrocyte sedimentation rate; BNP, brain natriuretic pepti.

Data are median (IQR) or *n*/ total (%). *P* value denotes the comparison among moderate, severe and critical illness group. * and †Signify *P* < 0.05 for post-hoc comparison. *Refers to comparison between the critical group and the severe group or the moderate group. †Refers to comparison between the severe group and the moderate group.

Totally, 320 (61.07%) patients were given antivirals within 2 days after admission including lopinavir/ritonavir, arbidol, darunavir and chloroquine. In all, 369 (70.83%) patients received antibiotics and 117 (23.08%) received corticosteroids. More patients received corticosteroids in critical group and severe group as compared with the moderate group (*P* < 0.05). The proportions of patients accepting high-flow nasal cannula oxygen therapy and non-invasive mechanical ventilation, respectively, in critical and severe groups were significantly higher than in mode rate group (*P* < 0.05). Compared with the moderate group, the critical group and the severe group had a significantly lower rate of discharge and a higher mortality rate (*P* < 0.05), as shown in Table 3. The comparison of demographic and baseline characteristics, symptoms, laboratory parameters, lung image features, treatment and prognosis among moderately, severely and critically ill patients were shown in Tables 1–3.

**Table 3.** Treatment and prognosis of 598 COVID-19 patients

Variable	All (*n* = 598)	Disease severity			*P* value
		Moderate (*n* = 400)	Severe (*n* = 85)	Critical (*n* = 113)	
Administration of antiviral	320/524 (61.07)	229/395 (57.97)*	42/62 (67.74)	49/67 (73.13)	0.032
Administration of antibiotics	369/521 (70.83)	253/398 (63.57)	52/58 (89.66)	64/65 (98.46)	<0.0001
Administration of corticosteroids	117/507 (23.08)	40/398 (10.05)*†	25/51 (49.02)*	49/58 (84.48)	<0.0001
Oxygen therapy					<0.0001
Nasal cannula or no oxygen therapy	459/583 (78.73)	394/399 (98.75)*†	45/79 (56.96)*	20/105 (19.05)	
High-flow nasal cannula	47/583 (8.06)	2/399 (5.01)*†	7/79 (8.86)*	38/105 (36.19)	
Non-invasive mechanical ventilation (face mask)	54/583 (9.26)	2/399 (5.01)*†	27/79 (24.18)	25/105 (23.81)	
Invasive mechanical ventilation	23/583 (3.95)	1/399 (2.51)*	0/79 (0.00)*	22/105 (20.95)	
Prognosis					<0.0001
Discharge	457/576 (76.42)	372/400 (93.00)*†	56/85(65.88)*	29/113 (25.66)	
Death	79/576 (13.21)	6/400 (1.50)*†	5/85 (5.88)*	68/113(60.18)	
Remained in hospital	62/576 (10.37)	22/400(5.50)*†	24/85 (28.24)	16/113 (14.16)	
Secondary viral infection	16/389 (4.11)	1/268 (0.37)*†	2/54 (3.70)*	13/67 (19.40)	<0.0001
Secondary bacterial infection	10/323 (3.10)	5/266 (1.88)†	4/40 (10.00)	1/17 (5.88)	0.027
Secondary fungal infection	9/307 (2.93)	8/263 (3.04)	1/27 (3.70)	0/17 (0.00)	0.756
Length of hospital stay – days	21 (13−39)	20 (13−35)†	29 (14−46)	23 (7−40)	0.041

Data are median (IQR) or *n*/ total (%). *P* value denotes the comparison among moderate, severe and critical illness group. * and †Signify *P* < 0.05 for post-hoc comparison. *Refers to comparison between the critical group and the severe group or the moderate group. †Refers to comparison between the severe group and the moderate group.

### Determinants of illness severity

Fifty-three variables on admission were successively included in the univariate ordinal logistic regression, and 35 variables were found to be associated with illness severity, including age, gender, hypertension, diabetes, interval between illness onset and diagnosis, interval between illness onset and admission, pharyngodynia, shortness of breath, early administration of antiviral, C-reactive protein, white blood cell (WBC) count, neutrophil count, lymphocyte count, haemoglobin, platelet count, ALT, AST, albumin, blood urea nitrogen, creatinine, potassium, LDH, creatine kinase, myohaemoglobin, troponin I (cTnI), PCT, erythrocyte sedimentation rate (ESR), BNP, fibrinogen, D-dimer, bilateral lungs involved, consolidation, linear opacity, pleural thickening and lung lobes involved (Table 4).

**Table 4.** Results of univariate ordinal logistic model using three levels of severity as response

Variable	Level	*β*	OR	95% CI	*P* value
*Demographics and clinical characteristics*					
Age (years)	<40	Ref			
	40–69	1.091	2.978	1.722–5.150	<0.0001
	⩾70	2.139	8.492	4.548–15.857	<0.0001
Gender	Male	0.408	1.503	1.064–2.124	0.0207
	Female	Ref			
Smoking status	Never	Ref			
	Current	−0.399	0.671	0.336–1.340	0.2581
	Former	−0.361	0.697	0.221–2.200	0.5387
Hypertension	Present	1.503	4.493	3.155–6.399	<0.0001
	Absent	Ref			
Cardiovascular disease	Present	0.117	1.124	0.574–2.199	0.7333
	Absent	Ref			
Diabetes	Present	0.772	2.164	1.363–3.437	0.0011
	Absent	Ref			
Carcinoma	Present	0.788	2.198	0.963–5.019	0.0614
	Absent	Ref			
COPD	Present	0.101	1.107	0.327–3.747	0.8708
	Absent	Ref			
Cerebrovascular disease	Present	0.148	1.159	0.458–2.931	0.7549
	Absent	Ref			
Fever	Absent	Ref			
	Present	0.041	1.042	0.653–1.662	0.8640
Dry cough	Absent	Ref			
	Present	0.038	1.039	0.726–1.487	0.8353
Sputum production	Absent	Ref			
	Present	−0.175	0.840	0.577–1.222	0.3609
Pharyngodynia	Absent	Ref			
	Present	−0.917	0.400	0.170–0.940	0.0356
Chest pain	Absent	Ref			
	Present	0.077	1.080	0.495–2.356	0.8459
Shortness of breath	Absent	Ref			
	Present	1.135	3.112	2.148–4.507	<0.0001
Early administration of antiviral	Present	Ref			
	Absent	0.465	1.592	1.138–2.227	0.0066
Interval between illness onset and diagnosis		0.079	1.082	1.048–1.116	<0.0001
Interval between illness onset and admission		0.110	1.116	1.083–1.150	<0.0001
*Laboratory findings*					
CRP (mg/l)	>100	1.813	6.126	3.400–11.039	<0.0001
	5–100	0.543	1.722	1.053–2.816	0.0304
	<5	Ref			
WBC count (×10^9^/l)	<4	Ref			
	4–10	0.186	1.204	0.735–1.973	0.460
	>10	1.228	3.416	1.834–6.360	0.0001
Neutrophil count (×10^9^/l)	>6.3	1.358	3.887	1.601–9.442	0.0027
	1.8–6.3	0.416	1.516	0.650–3.536	0.3360
	<1.8	Ref			
Lymphocyte count (×10^9^/l)	<0.5	0.987	2.684	1.508–4.776	0.0008
	0.5–1.1	0.449	1.567	1.096–2.239	0.0138
	⩾1.1	Ref			
Haemoglobin (g/l)	⩾130	Ref			
	<130	0.435	1.545	1.104–2.162	0.0112
Platelet count (×10^9^/l)	<125	Ref			
	125–350	0.315	1.370	0.802–2.341	0.2498
	>350	1.299	3.666	1.612–8.335	0.0019
ALT –(μ/l)	>50	1.331	3.783	2.593–5.521	<0.0001
	⩽50	Ref			
AST (μ/l)	>40	1.227	3.410	2.388–4.871	<0.0001
	⩽40	Ref			
Total bilirubin (mmol/l)	>20.5	−0.604	0.547	0.277–1.079	0.0817
	⩽20.5	Ref			
Direct bilirubin (mmol/l)	>8.6	0.051	1.052	0.590–1.875	0.8626
	⩽8.6	Ref			
Albumin (g/l)	<40	1.746	5.734	3.532–9.306	<0.0001
	⩾40	Ref			
Blood urea nitrogen (mmol/l)	>9.5	1.181	3.257	1.811–5.860	<0.0001
	⩽9.5	Ref			
Creatinine (μmol/l)	>111	1.293	3.645	1.927–6.895	<0.0001
	⩽111	Ref			
Urinary glucose	Positive	0.084	1.088	0.599–1.976	0.7827
	Negative	Ref			
Urinary protein	Positive	0.362	1.436	0.918–2.247	0.1133
	Negative	Ref			
Sodium (mmol/l)	>147	0.593	1.809	0.388–8.433	0.4506
	⩽147	Ref			
Potassium (mmol/l)	<3.5	Ref			
	3.5–5.3	1.046	2.847	1.459–5.557	0.0022
	>5.3	1.642	5.164	1.664–16.020	0.0045
Calcium (mmol/l)	⩾2.55	0.246	1.279	0.525–3.120	0.5882
	<2.55	Ref			
LDH (μ/l)	<245	Ref			
	⩾245	1.119	3.062	2.114–4.434	<0.0001
Creatine kinase (μ/l)	⩽200	Ref			
	>200	0.594	1.811	1.211–2.708	0.0038
CK–MB (μ/l)	⩽24	Ref			
	>24	−0.431	0.650	0.341–1.239	0.1905
Myohaemoglobin (ng/ml)	⩽48.8	Ref			
	>48.8	1.930	6.888	4.770–9.945	<0.0001
cTnI (ng/ml)	⩽0.04	Ref			
	>0.04	2.571	13.083	8.077–21.189	<0.0001
PCT (μ/l)	⩽0.05	Ref			
	>0.05	1.206	3.341	2.363–4.723	<0.0001
ESR (mm/h)	⩽15	0.631	1.879	1.084–3.258	0.0247
	>15	Ref			
BNP (pg/ml)	⩽250	Ref			
	>250	1.361	3900	2.479–6.136	<0.0001
Fibrinogen (g/l)	⩽4	Ref			
	>4	0.520	1.682	1.126–2.513	0.0111
D-dimer (μg/l)	⩽0.5	Ref			
	>0.5	1.266	3.548	2.414–5.214	<0.0001
*Lung imaging features*					
Bilateral lungs involved	Yes	1.286	3.618	1.519–8.616	0.0037
	No				
Consolidation	Yes	−0.977	0.319	0.201–0.704	0.0022
	No				
Ground-glass opacity	Yes	0.077	1.080	0.409–2.855	0.8766
	No				
Linear opacity	Yes	0.522	1.685	1.015–2.798	0.0437
	No				
Pleural effusion	Yes	0.139	1.149	0.439–3.008	0.7765
	No				
Pleural thickening	Yes	−0.510	0.600	0.375–0.961	0.0334
	No				
Lung lobes involved		0.426	1.531	1.270–1.847	<0.0001

COPD, chronic obstructive pulmonary disease; CRP, C-reactive protein; WBC, white blood cell; ALT, alanine aminotransferase; AST, aspartate aminotransferase; LDH, lactate dehydrogenase; CK-MB, creatine kinase isoenzymes; cTnI, cardiac troponin I; PCT, procalcitonin; ESR, erythrocyte sedimentation rate; BNP, brain natriuretic peptid.

Except five lung image variables, 30 significant predictors of severity in univariable analysis were included in a multivariable stepwise cumulative logit model, and seven variables retained in the final model which were statistically significant independent determinants of COVID-19 illness severity (Table 5). The results of multivariate model revealed that the risks of having more severe illness were 1.586 (95% CI: 0.824–3.053) and 3.419 (95% CI: 1.596–7.323) times higher among patients belonging to the age group 40–69 and 70+ years, respectively, when compared with patients of less than 40 years. Patients with hypertension had 3.372 (95% CI: 2.185–5.202) times greater risk of having worse severity of illness compared with patients without hypertension. The risk of having worse severity of illness was found higher for patients with ALT>50 μ/l (OR = 3.304; 95% CI: 2.107–5.180) when compared with those having ALT⩽50 μ/l. The risk of having worse severity of illness was found significantly higher for patients with higher cTnI (>0.04 ng/ml) than those with normal cTnI (⩽0.04 ng/ml) with OR being 7.464 (95% CI: 4.292–12.980). COVID-19 patients with myohaemoglobin>48.8 ng/ml at admission had a 2.214 (95% CI: 1.42–3.453)-fold greater risk of having worse severity of illness when comparison was made with patients having normal myohaemoglobin level. Table 5 also shows that interval between illness onset and diagnosis and interval between illness onset and admission were independent significant predictors of illness severity with OR being 1.056 (95% CI: 1.012–1.101) and 1.048 (95% CI: 1.009–1.087), respectively.

**Table 5.** Results of multiple ordinal logistic model using three levels of severity as response

Variable	Level	*β*	OR	95% CI	*P* value
Age (years)	<40	Ref			
	40–69	0.461	1.586	0.824–3.053	0.1675
	⩾70	1.229	3.419	1.596–7.323	0.0016
Hypertension	Present	1.215	3.372	2.185–5.202	<0.0001
	Absent	Ref			
ALT (μ/l)	>50	1.195	3.304	2.107–5.180	<0.0001
	⩽50	Ref			
cTnI (ng/ml)	⩽0.04	Ref			
	>0.04	2.010	7.464	4.292–12.980	<0.0001
Myohaemoglobin (ng/ml)	⩽48.8	Ref			
	>48.8	0.795	2.214	1.420–3.453	0.0005
Days between illness onset and diagnosis		0.054	1.056	1.012–1.101	0.0111
Days between illness onset and admission		0.047	1.048	1.009–1.087	0.0142

ALT, alanine aminotransferase; cTnI, cardiac troponin I.

### Post-hoc comparison of survival among patient groups with different risk factors

**Fig. 1.**
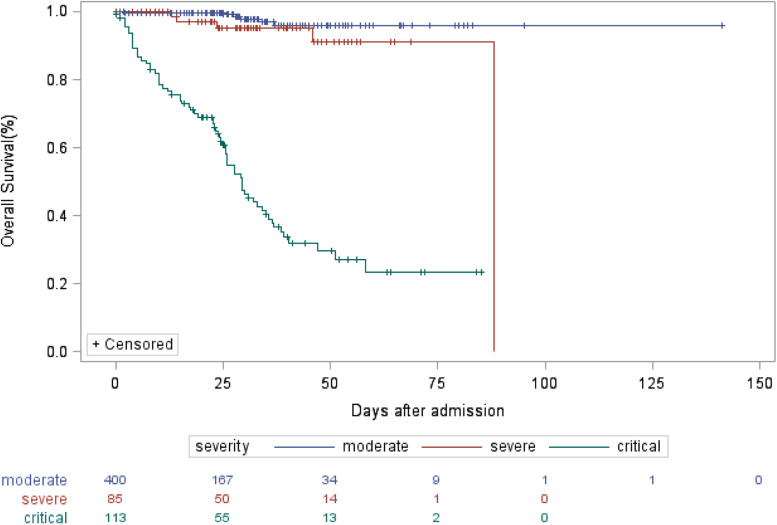
Kaplan−Meier estimate of OS of COVID-19 patients according to severity of illness.

In the critical illness group, 68 of 113 patients (60.18%) died, as compared with five of 85 (5.88%) in the severe illness group and six out of 400 (1.50%) in the moderate illness group. The median OS was 29 days in critical group, as compared with not attainable in severe group and moderate group. The 30-day OS rates were 97.7% (95% CI: 95.5–100%), 95.3% (95% CI: 90.2–100%) and 46.4% (95% CI: 37.4–57.6%) in moderate, severe and critical groups, respectively (*P* < 0.001), as shown in Figure 1. Patients of critical illness suffered from inferior survival, as compared with patients in severe group (HR = 14.309, 95% CI: 5.585–36.659) and those in moderate group (HR = 41.021, 95% CI: 17.588–95.678), representing 14.309 times greater risk of death and 41.021 times greater risk of death when compared with severe and moderate groups, respectively. As shown in Supplementary Table S2, COVID-19 patients of older age (HR = 9.823 for ⩾70 *vs.* <40; HR = 3.361 for 40–69 *vs.* <40), comorbidity of hypertension (HR = 3.161), abnormal ALT (HR = 1.657), abnormal cTnI (HR = 2.513) or abnormal myohaemoglobin (HR = 2.671) suffered from inferior survival. Also, the Kaplan−Meier survival curves (Supplementary Figs S1–S5) demonstrated that the differences of OS with respect to stratification by these risk factors were all statistically significant (*P* < 0.05).

## Discussion

COVID-19 has presented an unprecedented challenge to the health-care system across the world. With the increasingly scarce health resources, mortality is the most important issue when dealing with epidemics. Early identifying potential of severe and critical patients becomes the priority in minimising the mortality and contributes to allocation of limited critical care. Previously, a risk forecasting model to predict the occurrence of critical illness among hospitalised COVID-19 patients in China has been reported by Liang *et al*. [11]. The response variable in Liang's model had just two levels of severe and non-severe. Actually, the mortalities of COVID-19 patients with different severity are variant. This study demonstrated the mortality of 60.18% in critical cases, followed by 5.88% in severe cases and 1.50% in moderate cases. Kaplan−Meier analysis also showed that patients of critical illness suffered from inferior survival, as compared with patients of severe illness and moderate illness, indicating that illness severity is related to the prognosis. In our opinion, more precise classification of illness severity into three levels suits the clinical spectrum of COVID-19 and the predicting model based on multi-level ordinal response variables can be more practical. The current multi-centre retrospective study identified the risk factors of illness severity among COVID-19 inpatients from four hospitals of Hubei, Anhui and Shanghai. According to the Guideline on the Diagnosis and Treatment of COVID-19 by the National Health Commission, inpatients of COVID-19 in this study were divided into moderately ill, severely ill and critically ill groups, and the ordinal logistic regression model was fitted to identify the predicators of severity of illness. Based on current data, older age, comorbidity of hypertension, elevated levels of ALT, elevated cTnI, elevated myohaemoglobin, together with prolonged interval between illness onset and diagnosis and interval between illness onset and admission were independent determinants of severity of COVID-19 and represented higher odds of worse severity of illness.

Evidence is gradually accumulating with regard to the risk factors associated with severity of COVID-19. As described previously, older age has been reported as an important independent predictor of severity in COVID-19 patients [5, 6, 11], which is proved by this study. Comorbidity of hypertension was found to be associated with an increased risk of death. A meta-analysis including 46 248 patients with confirmed COVID-19 indicated that those with the most severe illness were more likely to have hypertension with OR of 2.36 (95% CI: 1.46–3.83). Hypertension was reported to increase the OR for death by 3.05 (95% CI: 1.57–5.92) in patients with COVID-19 [4]. Similarly, patients with hypertension had 3.372 (95% CI: 2.185–5.202) times greater risk of developing worse severity of illness in comparison with those without hypertension in our study. The relationship between hypertension and COVID-19 may relate to the role of angiotensin converting enzyme (ACE2) [13]. As a key element in the renin–angiotensin–aldosterone system (RAAS), ACE2 is critically involved in the pathophysiology of hypertension. Studies demonstrated that inhibition of the RAAS with ACE inhibitors (ACEIs) or angiotensin II receptor blockers (ARBs) may result in a compensatory increase in tissue levels of ACE2 and poorer clinical course and prognosis, leading to suggestions that these drugs may be detrimental to COVID-19 patients [14].

It has been reported that COVID-19 had significant impact on the liver function [15, 16]. A meta-analysis including 20 retrospective studies with 3428 COVID-19 patients revealed that higher serum levels of AST (mean difference = 8.84 U/l, 95% CI: 5.97–11.71, *P* < 0.001) and ALT (mean difference = 7.35 U/l, 95% CI: 4.77–9.93, *P* < 0.001) and lower serum levels of albumin (mean difference = −4.24 g/l, 95% CI: −6.20 to −2.28, *P* < 0.001) were associated with a significant increase in the severity of COVID-19 [17]. Our univariate logistic regression results also showed patients with elevated AST, ALT and decreased albumin had 3.410-, 3.783- and 5.734-fold greater risk of worse severity, which was consistent with this meta-analysis. Particularly, our multivariate ordinal regression demonstrated that ALT is an independent predictor of severity of COVID-19 patients. Since elevated liver injury indicators are strongly associated with the severity risk and subsequent death risk, the liver function should be monitored during hospitalisation. Acute myocardial injury is the most commonly described cardiovascular complication in COVID-19 [18]. The overall incidence of acute myocardial injury has been variable but roughly 8–12% of COVID-19 patients are found to develop significant elevation of cTnI [19]. The patients admitted to ICU or having severe/fatal illness have several-fold higher likelihood of cTnI elevation. Several recent studies indicated that higher concentration of cTnI and myohaemoglobin were associated with the severity and case fatality rate of COVID-19 [20–22]. Chen *et al*. reported that elevated cTnI (OR = 26.909, 95%CI: 4.086–177.226, *P* = 0.001) were the independent risk factors of critical disease status [20]. Han *et al*., found there were statistically significant differences in the level and positive rate of cTnI and myohaemoglobin among the mild, severe and critical COVID-19 case groups [23]. Our results are in agreement with the previous studies that the elevated myocardial injury markers such as cTnI and myohaemoglobin are independent determinants of illness severity in COVID-19 patients representing negative clinical course and potentially life-threatening prognosis. In particular, cTnI is the strongest predictor of worse severity with OR being 7.464 (95% CI: 4.292–12.980). Direct myocardial injury due to viral myocarditis or the effect of systemic inflammation appears to be the most common mechanisms of acute cardiac injury. Our study also found that interval between illness onset and diagnosis and interval between illness onset and admission were independently associated with illness severity. The risk of developing worse illness severity increased by 1.056-fold and 1.048-fold for each day delay of interval between illness onset and diagnosis and interval between illness onset and admission, respectively. Chen *et al*. [24] found the median time from symptom onset to admission was 10 days among deceased COVID-19 patients, one day longer than that of discharged patients. Considering the limited health resources and high case volume in some regions currently, adjusted tactics and strategies should be taken to maximise the availability of and accessibility to medical service to shorten the diagnosis delay or admission delay.

Our study had several limitations. First, this is a retrospective study design, which could be subject to recall bias and selection bias. Second, not all laboratory parameters were tested in all patients, including LDH and D-dimer. Although imputation technique was used to replace missing values, their role might be underestimated in predicting illness severity. Third, due to massive loss of chest CT results, the predicting role of chest CT abnormalities could not be evaluated in this study. Its role of predicting critical illness of COVID-19 has been demonstrated by other studies. Last but not least, generalisability of our findings might be limited by the sample size, and the results need to be validated based on a much larger patient population.

In this study, we identified older age, presence of hypertension, elevated ALT, cTnI and myohaemoglobin, prolonged interval between illness onset and diagnosis and admission as the independent determinants to predict the risk of developing more severe illness among COVID-19 patients. Given the ongoing global pandemic of COVID-19, this study will contribute to early identifications of patients with high risk of developing critical illness and optimising the arrangement of health resources.

## Data Availability

The data that support the findings of this study are available from Clinical Research Center, Ruijin Hospital, Shanghai Jiao Tong University School of Medicine. Restrictions apply to the availability of these data, which were used under license for this study.

## Supplementary material

For supplementary material accompanying this paper visit http://dx.doi.org/10.1017/S0950268820001533. click here to view supplementary material
