# Supplementary material for: Application of ordinal logistic regression analysis to identify the determinants of illness severity of COVID-19 in China
Source: Epidemiol Infect. 2020 Jul 7;148:e146. doi: 10.1017/S0950268820001533 (PMC7369341; doi:10.1017/S0950268820001533)
Supplement: Supplementary file 1 [file S0950268820001533sup001.docx]

**Supplementary Table 1:** Reference range of laboratory indicators

| Indicator | Unit | Reference range |
| --- | --- | --- |
| C-reactive protein | mg/L | <5.00 |
| White blood cell count | ×10^9^/L | 4.00~10.00 |
| Neutrophil count | ×10^9^/L | 1.80~6.30 |
| Lymphocyte count | ×10^9^/L | 1.10~3.20 |
| Haemoglobin | g/L | 130.00~175.00 |
| Platelet count | ×10^9^/L | 125~350 |
| ALT | u/L | 9.0~50.0 |
| AST | u/L | 15.0~40.0 |
| Total bilirubin | umol/L | 3.40~20.50 |
| Direct bilirubin | umol/L | 0~8.60 |
| Albumin | g/L | 40.00~55.00 |
| Blood urea nitrogen | mmol/L | 3.60~9.50 |
| Creatinine | umol/L | 57.00~111.00 |
| Sodium | mmol/L | 137.00~147.00 |
| Potassium | mmol/L | 3.50~5.30 |
| Calcium | mmol/L | 2.10~2.55 |
| LDH | u/L | 109.00~245.00 |
| Creatine Kinase | u/L | 30.00~200.00 |
| CK-MB | u/L | <24 |
| Myohemoglobin | ng/ml | <48.80 |
| cTnI | ng/ml | <0.04 |
| PCT | ug/L | <0.05 |
| ESR | mm/h | <15 |
| BNP | pg/ml | <250 |
| Fibrinogen | g/L | 2.00~4.00 |
| D-dimer | ug/L | <0.50 |

**Supplementary Table 2:** Cox regression analysis of factors associated with overall survival of COVID-19 patients

| Variable | Level | Unadjusted HR | 95%CI | *P* value |
| --- | --- | --- | --- | --- |
| Age (years) | <40 | Ref |  |  |
|  | 40-69 | 3.361 | 1.193-9.468 | 0.0218 |
|  | ≥70 | 9.823 | 3.402-28.365 | <0.0001 |
| Hypertension | Yes | 3.161 | 1.998-4.999 | <0.0001 |
|  | No | Ref |  |  |
| ALT - u/L | >40 | 1.657 | 1.051-2.614 | 0.0298 |
|  | ≤40 | Ref |  |  |
| Troponin I- ng/ml | ≤0.04 | Ref |  |  |
|  | >0.04 | 2.513 | 1.397-4.518 | 0.0021 |
| Myohemoglobin - ng/ml | ≤48.8 | Ref |  |  |
|  | >48.8 | 2.671 | 1.638-4.355 | <0.0001 |


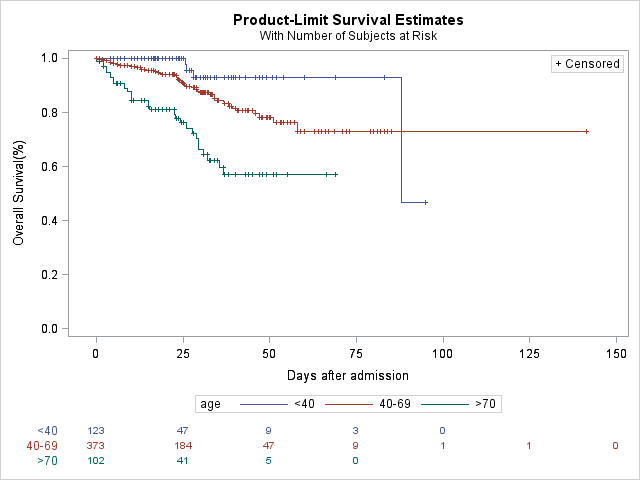


**Supplementary Figure 1:** Kaplan-Meier estimate of overall survival of COVID-19 patients according to age group


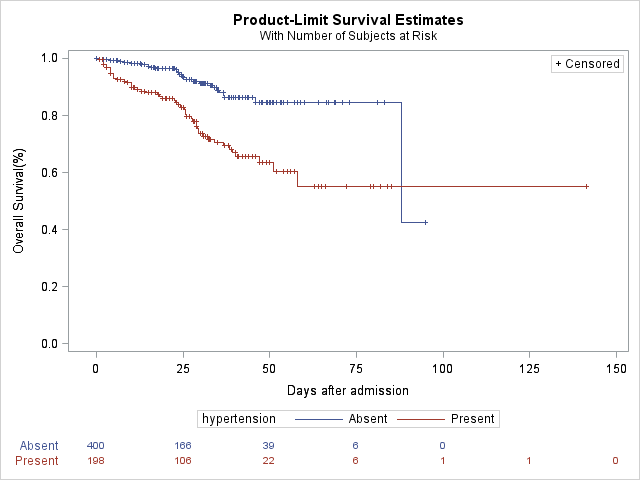


**Supplementary Figure 2:** Kaplan-Meier estimate of overall survival of COVID-19 patients according to comorbidity of hypertension


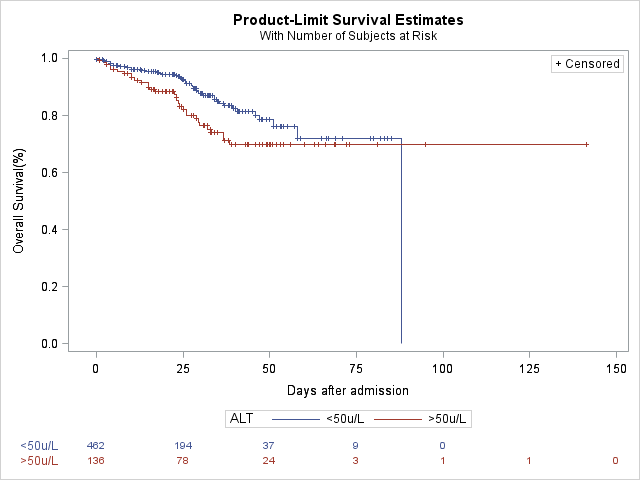


**Supplementary Figure 3:** Kaplan-Meier estimate of overall survival of COVID-19 patients according to level of ALT


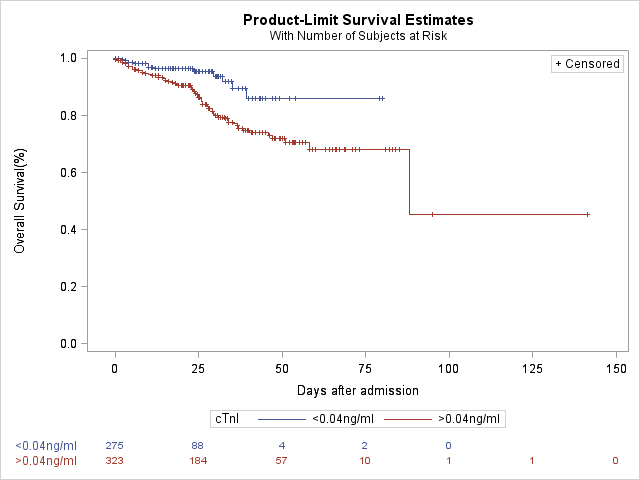


**Supplementary Figure 4:** Kaplan-Meier estimate of overall survival of COVID-19 patients according to level of cTnI


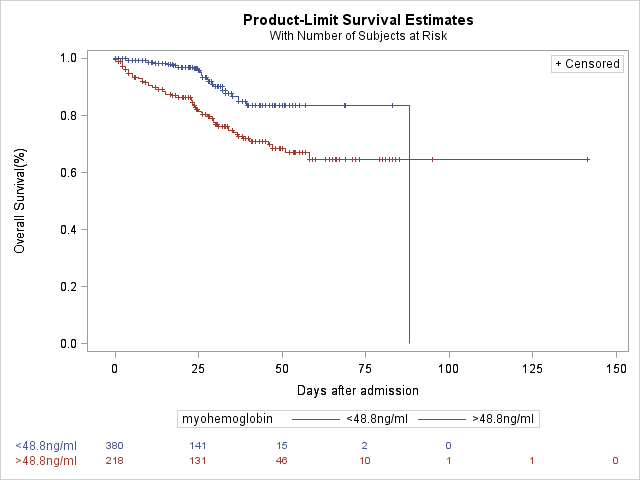


**Supplementary Figure 5:** Kaplan-Meier estimate of overall survival of COVID-19 patients according to level of myohemoglobin
